# Cardiomyopathy and mitochondrial encephalomyopathy in a female child associated with a heterozygous X-linked AIFM1 variant

**DOI:** 10.1186/s40348-026-00246-z

**Published:** 2026-06-22

**Authors:** Christoph Sandmann, Swathi Gudapati, Johannes A. Mayr, Rami Abou Jamra, Alexander Kovacevic, Steffen Syrbe

**Affiliations:** 1 Department of Genetics, Harvard Medical School, Boston, MA USA; 2 Department of Pediatric and Congenital Cardiology, Heidelberg University Hospital, Heidelberg, Germany; 3 University Children’s Hospital, Paracelsus Medical University, Salzburg, Austria; 4 Institute of Human Genetics, Paracelsus Medical University, Salzburg, Austria; 5 Institute of Human Genetics, University of Leipzig Medical Center, Leipzig, Germany; 6 Division of Pediatric Epileptology, Medical Faculty of Heidelberg, Center for Child and Adolescent Medicine, Clinic 1, Heidelberg University Hospital, Heidelberg, Germany

**Keywords:** Cardiomyopathy, Mitochondrial Encephalomyopathies, Mitochondrial diseases, Inborn, Rare diseases, Genetic diseases, Pediatrics

## Abstract

**Background:**

*AIFM1* encodes the X-linked oxidoreductase ‘apoptosis-inducing factor 1, mitochondrial’ that mediates caspase-independent programmed cell death and is involved in redox metabolism. To date, cardiac involvement has been reported in four patients with *AIFM1* variants, primarily presenting as ventricular hypertrophy, but its clinical course and prognosis remain not well understood.

**Methods:**

We report the first affected female with a heterozygous *AIFM1* variant who developed infantile-onset mitochondrial encephalomyopathy and cardiomyopathy with initial ventricular hypertrophy, that progressed to left ventricular dilation and chronic heart failure. In addition, we review the available literature on *AIFM1*-associated cardiomyopathy to contextualize clinical findings.

**Results:**

Genetic testing identified a heterozygous *AIFM1* variant, c.506C > T (p.Pro169Leu), with extremely skewed X-inactivation (98:2) in a female. The patient presented with infantile-onset mitochondrial encephalomyopathy. Echocardiography at 8 months revealed marked left ventricular hypertrophy with preserved systolic function. During follow-up, the cardiac phenotype progressively evolved into dilated cardiomyopathy with systolic dysfunction by 2.5 years of age, necessitating initiation of heart failure therapy.

**Conclusions:**

A heterozygous *AIFM1* variant can result in disease manifestation in females. The phenotypic spectrum of *AIFM1*-related disease includes cardiomyopathy, typically characterized by early-onset cardiac hypertrophy that may progress to ventricular dilatation and heart failure. This case highlights the importance of early recognition and careful cardiac monitoring in affected individuals, including female variant carriers.

**Supplementary Information:**

The online version contains supplementary material available at 10.1186/s40348-026-00246-z.

## Introduction

The X-linked gene *AIFM1* encodes apoptosis-inducing factor, mitochondrial 1, a NADH-binding flavin adenine dinucleotide (FAD)-dependent oxidoreductase in the mitochondrial intermembrane space with essential roles for mitochondrial function, redox control, and non-caspase-dependent cell death [1, 2]. Pathogenic variants in *AIFM1* are associated with a broad and heterogenous spectrum of clinical phenotypes that show a phenotypical overlap with other mitochondriopathies, such as mitochondrial encephalomyopathy, axonal sensorimotor neuropathy, ataxia or deafness. Specific phenotypes described include X-linked mitochondrial encephalomyopathy (COXPD6), Cowchock syndrome, X-linked deafness-5, spondyloepimetaphyseal dysplasia with hypomyelinating leukodystrophy, early-onset hearing loss with progressive cerebellar ataxia and an infantile SMA-like motor neuron disease. Mitochondrial disease is often accompanied by cardiomyopathy, which may adversely affect prognosis [3, 4], however the cardiac involvement in *AIFM1*-related disease remains incompletely described. Its clinical course, risk of progression, and long-term prognosis remain unknown. To the best of our knowledge, pathogenic cardiac manifestation of 4 cases with *AIFM1* variants were previously reported [5–7]. In mice, muscle-specific loss of *Aifm1* causes severe dilated cardiomyopathy, heart failure, and skeletal muscle atrophy accompanied by lactic acidemia consistent with defects in the mitochondrial respiratory chain [8].

Here, we describe the longitudinal follow-up of a female child carrying a de novo heterozygous *AIFM1* variant c.506C >T (p.Pro169Leu), who developed mitochondrial encephalomyopathy and childhood-onset cardiomyopathy, initially presenting with left ventricular hypertrophy and subsequently evolving into cardiac dilatation with heart failure. This case expands the phenotypic spectrum of *AIFM1*-associated disease by demonstrating clinical manifestations in a heterozygous female variant carrier. By providing a detailed clinical course together with a description and review of *AIFM1*-related cardiomyopathy, we highlight the importance of systematic cardiac surveillance in affected individuals, potentially including female carriers of this X-linked disorder.

## Case report

A female infant was delivered at 39 + 0 weeks of gestation via planned primary caesarean section, weighing 2440 g and measuring 47 cm, with APGAR scores of 9, 10, and 10. Early pregnancy ultrasound revealed increased nuchal translucency, but chorionic villus sampling excluded chromosomal abnormalities. The mother had been taking levothyroxine (50 µg/day) throughout pregnancy. Uterine artery Doppler showed mildly elevated vascular resistance; otherwise, the pregnancy was uneventful. There were no perinatal complications. The patient has one healthy older brother, and there is no family history of neurological, cardiac, or metabolic disorders.

**Fig. 1:**
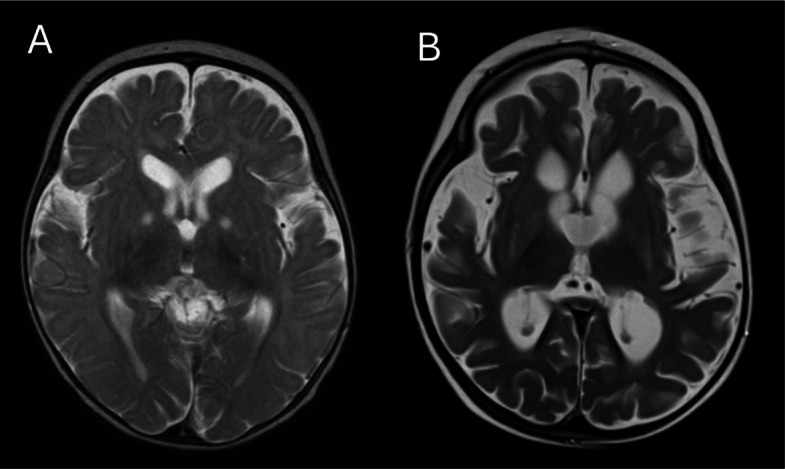
Representative brain MRI at 7 and 20 months of age. Serial MRI-brain imaging at 7 (**A**) and 20 (**B**) months of age, showing progressive changes with abnormal myelination, T2-hyperintense pallidal lesions and progressive atrophy

The patient was first admitted to our department at 7 months of age for evaluation of suspected West syndrome, developmental regression, and loss of social interaction. According to the parents, symptoms began around 2 months of age, including poor feeding, frequent crying episodes, and reduced responsiveness. Clinical features initially worsened during a transient period of increased irritability lasting 1–2 months, followed by decreased vocalization, loss of social smiling, and stagnation in weight gain. The mother reported seizure-like episodes from early infancy, occurring approximately six times a day, characterized by sudden forward flexion of the head and torso, adduction of the arms, absence of crying or cyanosis, and subsequent sleep. Additionally, the parents noted a musty odor of the urine and sticky, abnormally odorous stools. Physical examination revealed generalized muscular hypotonia, microcephaly (head circumference 39.8 cm, < 1 st percentile, SDS −4.07) and growth retardation (height 62.5 cm, < 1 st percentile, SDS −2.92; weight 5.71 kg (1st percentile, SDS −2.27); BMI 14.62 kg/m^2^ (9th percentile, SDS −1.36); BSA 0.31 m^2^. Electroencephalography (EEG) demonstrated hypsarrhythmia without focal abnormalities, confirming the diagnosis of West syndrome. Further testing revealed elevated lactate in both blood (31.0 mg/dl, norm. < 16 mg/dl) and cerebrospinal fluid (4.00 mmol/l, norm. 1.1–1.8 mmol/l), mildly increased cerebrospinal fluid protein, and high alanine levels. Urine analysis showed elevated lactate/creatinine ratios and mitochondrial metabolites, specifically of 2-Methyl-2,3-dihydroxy-butyrate, 3-hydroxy-methylglutaconate and 3-hydroxy-glutaric acid. Serial brain magnetic resonance imaging demonstrated progressive structural abnormalities characteristic of mitochondrial encephalopathy, including abnormal myelination, round T2-hyperintens pallidal lesions, deep grey matter involvement and cortical atrophy (Fig. 1).

Overall, the patient’s presentation was consistent with a mitochondrial encephalomyopathy. Genetic testing at the age of 8 months subsequently identified a de novo *AIFM1* (GenBank NM_004208.4) variant, c.506C>T, p.(Pro169Leu). Considering X-linked recessive inheritance, further genetic diagnostics additionally ruled out variation of mitochondrial DNA.

To assess the impact of the variant on mitochondrial function, we measured enzyme activity in patient’s fibroblasts. The analyzed mitochondrial respiratory chain enzymes: complex I, complex I + III, complex II, complex II + III, complex III, complex V, and pyruvate dehydrogenase were all normal in relation to protein content, marker enzyme citrate synthase and cytochrome c oxidase. (Supplementary Data 1). Additionally, X-inactivation was assessed and showed extremely skewed inactivation with a pattern 98:2, supporting the presence of an X-linked disorder. Therefore, the de novo *AIFM1* variant was considered causative for the girl’s phenotype despite normal mitochondrial respiratory chain enzyme activities.

Initial antiepileptic therapy with prednisolone and vigabatrin led to temporary seizure and EEG improvement, however epileptic spasms recurred during prednisolone tapering leading to add-on treatment with levetiracetam, gabapentin, lacosamide, phenobarbital and ketogenic diet. With suspicion of a mitochondrial disorder pyridoxine, thiamine, riboflavin, and coenzyme Q were supplemented. EEG improved slightly but remained pathological, with ongoing seizures. During this period, recurrent daytime and nighttime bradycardia was observed and electrocardiogram (ECG) evaluation was initiated at the age of 7.5 months. Resting 12-lead ECG and 24-h Holter ECG showed a regular sinus rhythm without pathological ECG intervals or arrythmia. Auditory testing revealed bilateral Eustachian tube dysfunction on brainstem-evoked response audiometry.

**Fig. 2:**
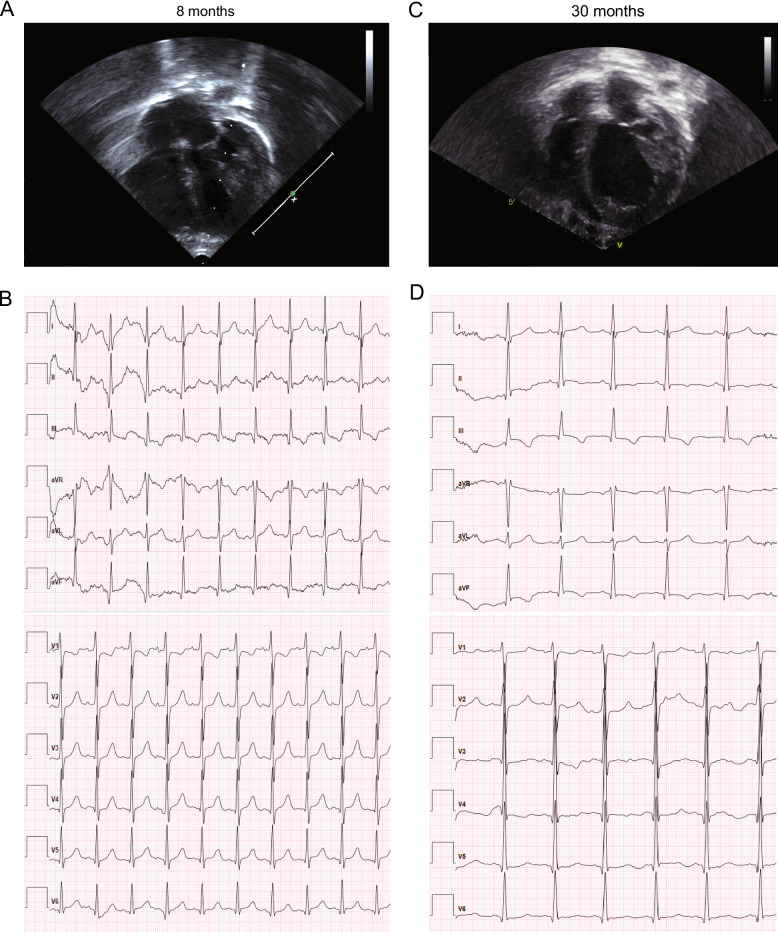
Representative echocardiographic and electrocardiographic findings at 8 and 30 months of age. **A** and **B**, Representative echocardiographic image, 4-chamber-view, (A) and electrocardiogram (B) of the cardiac phenotype at 8 months. **C** and **D**, Representative echocardiographic image, 4-chamber-view, (C) and electrocardiogram (D) of the cardiac phenotype at 30 months

Shortly after a discharge, the patient was readmitted due to declining general condition and recurrent feeding difficulties. Although no clinical seizures were observed, therapy was adjusted with further reduction of prednisolone, increased levetiracetam, gradual titration of vigabatrin, and initiation of a ketogenic diet, resulting in clinical stabilization. At the age of 8 months, echocardiography revealed left ventricular hypertrophy (IVSd z-score + 3.5, LVPWd z-score + 3.6) without outflow tract obstruction and with preserved left ventricular systolic function (FS 42.4%, EF 76.2%) (Fig. 2A). This was initially interpreted as a potential phenotype of hypertrophic cardiomyopathy in view of the underlying diagnosis. The ECG did not show any major repolarization abnormalities or arrhythmia (Fig. 2B). At the age of 11 months, the patient remained stable, exhibiting daily myoclonic jerks and 2–3 generalized seizures per week, which required interruption. Feeding had previously been via nasogastric tube due to dysphagia and was later transitioned to a percutaneous endoscopic gastrostomy. Echocardiographic findings remained stable.

By 13 months of age, ongoing spasms, gelastic seizures and myoclonia were observed. She developed dystonic movements and urinary retention. Clonidine was added to reduce dystonia motor restlessness. Chloral hydrate and midazolam were administered for sleeping disorder. Polypharmacy, cofactor substitution and ketogenic diet may have led to overall clinical stabilization, and EEG improvement, with only brief myoclonic episodes and occasional hypermotor episodes.

Over time, progressive left ventricular dilation and systolic dysfunction occurred. By the age of approximately 1.5 years, myocardial hypertrophy remained stable and ventricular dilation was observed, with a LVEDd z-score of + 3.9, and EF had declined to 51.2% (FS 25.6%). At 2 years and 5 months, bilateral neurogenic hip subluxation was diagnosed. Despite occasional respiratory infections and ongoing motor episodes, the overall condition remained stable, with levetiracetam adjusted to four daily doses. At the age of 2 years and 6 months the diagnostic criteria for pediatric dilative cardiomyopathy were fulfilled (LVEDd z-score + 4.4, FS 16,9%, EF 35.3%) despite persistent left ventricular hypertrophy (Fig. 2C). An ECG demonstrated left ventricular hypertrophy with terminal repolarization abnormalities in the inferolateral leads (Fig. 2D). At this point, heart failure therapy was initiated with good tolerance of low-dose beta-blocker (propranolol) and ACE inhibitor (ramipril). Follow-up imaging at the age of 4 years showed partial stabilization with improved left ventricular systolic function (FS 21.4%, EF 44.4%) and reduced left ventricular dilatation while still showing left ventricular hypertrophy. During the further course spironolactone and furosemide were added to the cardiac therapy.

**Fig. 3:**
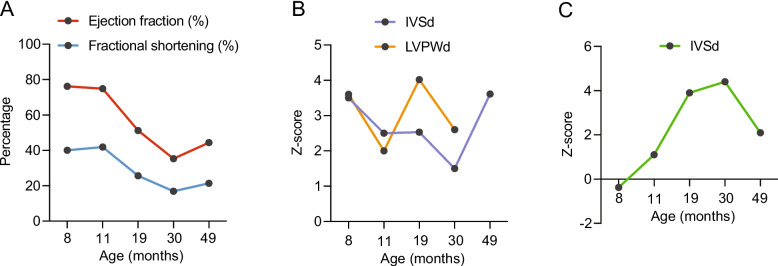
Longitudinal progression of the cardiac phenotype. **A **to **C** Longitudinal progression of echocardiogrohic parameters. IVSd (interventricular septum in diastole), LVPWd (left ventricular posterior wall in diastole), LVIDd (left ventricular internal dimension in diastole)

At 6 years she remained non-ambulant with quadriplegia, non-verbal with dysphagia, requiring tube feeding. Dystonia and occasional seizures are ongoing. Clinical course of the cardiac phenotype is summarised in Fig. 3. Pharmacological therapy is summarized in Supplementary Table 1.

## Discussion

Mitochondrial diseases, with an estimated prevalence of 1 in 5,000 births, comprise a heterogeneous group of metabolic disorders that predominantly manifest during infancy and may present as systemic or tissue-specific pathologies [9]. The underlying cause is typically congenital or progressive impairment of mitochondrial respiratory chain activity, often associated with severe neuromuscular phenotypes [9]. Mitochondrial diseases are commonly associated with cardiomyopathies, often presenting as severe hypertrophic or dilative cardiomyopathies that manifest during early childhood [3, 4]. A substantial proportion of pediatric cardiomyopathies are caused by mitochondrial disorders. However, when cardiac manifestations predominate at presentation, the underlying mitochondrial etiology may be overlooked, as analysis of the mitochondrial genome is not routinely included in first-line genetic testing for pediatric cardiomyopathies [3, 10].

Muscle or affected-organ biopsy with respiratory chain enzyme analysis and electron microscopy has traditionally been regarded as a key diagnostic approach for mitochondrial disease. A definitive diagnosis of mitochondrial disease typically requires evidence of impaired respiratory chain complex activity in biopsy specimens from the affected organ, abnormal findings on electron microscopy, and the presence of a pathogenic genetic mutation. However, invasive biopsy procedures are often not performed in infancy. In addition, normal respiratory chain complex activity in fibroblasts does not exclude mitochondrial disease because of tissue-specific heterogeneity. In patients with suspected mitochondrial disease, current diagnostic evaluation generally includes clinical assessment for multisystem involvement, metabolic screening, neuroimaging and organ-specific investigations, genetic testing, and, when required, functional validation studies. In cases involving novel variants or atypical phenotypes, diagnosis should rely on a comprehensive approach integrating clinical manifestations, metabolic findings, neuroimaging, genetic analyses, segregation studies, and functional validation studies whenever feasible, however, those may be especially challenging in infants. Recently, functional assays such as oxygen consumption rate analysis using extracellular flux analyzers have emerged as useful complementary tools for evaluating mitochondrial function. In the present case, the diagnosis was considered highly likely because the patient demonstrated a characteristic multisystem phenotype together with a previously reported pathogenic AIFM1 variant.

*AIFM1* encodes ‘Apoptosis-inducing factor 1, mitochondrial’, a membrane bound NADH-binding flavin adenine dinucleotide (FAD)-dependent oxidoreductase located in the mitochondrial intermembrane space [2]. AIFM1 plays an essential role in mitochondrial function by regulating the biogenesis and activity of respiratory chain complexes [2, 11–13]. This function is mediated indirectly through its redox-regulated role in facilitating the import of the oxidoreductase MIA40 (also known as CHCHD4) into the intermembrane space, which in turn governs the import and assembly of respiratory chain subunits [2, 14, 15]. Additional functions of AIFM1, potentially independent of MIA40, have also been proposed in the regulation of mitochondrial homeostasis [1, 16]. Pathogenic variants in *AIFM1* impair oxidative phosphorylation, resulting in decreased respiratory chain complex activity and reduced metabolic capacity in affected patients [17, 18]. In addition, *AIFM1* is a well characterized mediator of caspase-independent apoptosis [19]. Disturbance of which molecular function of AIFM1 causes the clinical phenotype is unknown. Potential mechanisms include reduced AIFM1 expression resulting in impaired mitochondrial function, as carriers of *AIFM1* variants have been shown to exhibit reduced respiratory chain complex activity [7, 18, 20].

To the best of our knowledge, cardiac involvement in *AIFM1*-related disease has been reported in four male children. Berger et al. (2011) described a male infant carrying a hemizygous c.923G>A, (p.Gly308Glu) variant who died at 3 months of age from hypertrophic cardiomyopathy and aspiration pneumonia [5]. Heimer et al. (2018) reported a 10.5-year-old male with a hemizygous c.422C>T (p.Thr141Ile) variant who presented with cardiomyopathy characterized by mild left ventricular dilatation and borderline systolic function. Moss et al. (2021) described two related male patients: one, in whom genetic testing confirmed a hemizygous c.506C>T (p.Pro169Leu) variant, presented with biventricular myocardial hypertrophy detected by foetal echocardiography; the other died at 39 days of life, with autopsy revealing biventricular cardiac hypertrophy. All four patients exhibited neurological manifestations, and three of the four presented with severe infantile-onset multisystem disease.

Our patient carries the same *AIFM1* c.506C>T (p.Pro169Leu) variant previously reported by Moss et al. and represents, to the best of our knowledge, the first clinically affected female child described to date. Moss et al. (2021) reported normal pyruvate dehydrogenase complex activity in cultured skin fibroblasts, which is consistent with the normal pyruvate dehydrogenase complex activity observed in our patient’s fibroblasts. While pyruvate dehydrogenase complex activity was also normal in skeletal muscle, Moss et al. identified reduced complex IV (cytochrome c oxidase) activity in muscle, whereas complex IV activity was normal in our patient’s fibroblasts. These findings suggest potential tissue-specific differences between fibroblasts and muscle, or that the phenotype may develop independently of measurable defects in mitochondrial enzyme activity, possibly through mechanisms of AIFM1 that are not directly related to respiratory chain metabolism. Nevertheless, our patient presented with elevated lactate levels in both blood and cerebrospinal fluid, increased alanine levels, and elevated mitochondrial metabolites of 2-Methyl-2,3-dihydroxy-butyrate, 3-hydroxy-methylglutaconate and 3-hydroxy-glutaric acid in urine, findings that are consistent with impaired oxidative metabolism and suggest an underlying mitochondrial dysfunction. Interestingly, the mother of the affected males described by Moss et al. (2021) was not affected by symptoms of the heart and brain despite being carrier of the germline variant. Extreme skewed X-inactivation and the typical brain abnormalities on MRI support the causality of the *AIFM1* variant for the phenotype in our girl with a progressive neurological phenotype with cerebral atrophy. While sharing core features with the previously reported male cases, including hypotonia, West syndrome, developmental regression, lactic acidosis, and mitochondrial dysfunction, her disease course was later in onset (approximately 2 months of age) and more slowly progressive. Cardiac involvement first became apparent at 7 months, initially presenting as ventricular hypertrophy, which evolved into cardiac dilatation by 1.5 years of age, but partially improved under heart failure therapy by the age of 3 years. This difference in severity is consistent with the X-linked inheritance pattern of *AIFM1*-related disease. With antiepileptic therapy and a ketogenic diet, clinical stabilization was achieved, and she remains alive beyond 6 years of age. Riboflavin was shown to improve mitochondrial dysfunction in a patient carrying a pathogenic *AIFM1* variant, resulting in clinical stabilization [21] and was early introduced into her treatment.

Additional mechanistic support for the understanding of the cardiac phenotype in our patient comes from a muscle specific *Aifm1* knockout mouse model, in which loss of AIF function led to mitochondrial dysfunction, skeletal muscle atrophy and early-onset dilated cardiomyopathy [8]. The mutant mice developed cardiac hypertrophy and severely reduced contractile function within weeks, closely mirroring the initial hypertrophy and systolic dysfunction observed in our case. Functional studies revealed significant complex 1 deficiency in mutant cardiac muscle tissue, lactic acidosis, metabolic shifts towards glycolysis and damage in mitochondrial structure. Out of these, only lactic acidosis was present in our patient. The remaining findings are pathophysiologically plausible but couldn’t be confirmed due to lack of tissue studies. Similarly, diminished *Aifm1* expression was observed in the cardiac tissue of the Harlequin mouse mutant, rendering the myocardium more susceptible to oxidative stress and pressure overload [22]. This animal models provides strong preclinical evidence that *AIFM1* is essential for cardiac mitochondrial function, with its loss directly resulting in progressive heart failure.

Our case highlights the importance of considering cardiac involvement in patients with *AIFM1* variants, even in females, where symptoms may present later and progress more slowly. Early cardiac screening including echocardiography and biomarker analysis (e.g. lactate levels, NT-proBNP) should be part of the diagnostics in suspected cases. Timely initiation of heart failure therapy in addition to co-factor treatment may lead to partial functional recovery.

## Supplementary Information

Supplementary Material 1: Supplementary Data 1: mitochondrial enzyme activity report.

Supplementary Material 2.

## Data Availability

No datasets were generated or analysed during the current study.
