# Supplementary material for: Cardiomyopathy and mitochondrial encephalomyopathy in a female child associated with a heterozygous X-linked AIFM1 variant
Source: Mol Cell Pediatr. 2026 Jun 22;13:34. doi: 10.1186/s40348-026-00246-z (PMC13287274; doi:10.1186/s40348-026-00246-z)
Supplement: Supplementary file 1 — Supplementary Material 1: Supplementary Data 1: mitochondrial enzyme activity report. [file 40348_2026_246_MOESM1_ESM.docx]

# Supplementary Data 1: Mitochondrial Enzyme Activity Report

## 1) Absolute Activities (mU/mg protein)

| **Enzyme / Complex** | **Iso 1** | **Iso 2** | **Reference Range** | |
| --- | --- | --- | --- | --- |
| Citrate Synthase (CS) | 219 | 217 | 225–459 | |
| Complex I | 18 | 23 | 18–53 | |
| Complex I+III | 224+ | 112 | 61–220 |  |
| Complex II | 62 | 125+ | 54–124 | |
| Complex II+III | 139 | 298+ | 79–219 | |
| Complex III | 654 | 1171+ | 208–648 | |
| Cytochrome c Oxidase (COX) | 411 | 545 | 270–659 | |
| Complex V | 237 | 315+ | 78–287 | |
| Pyruvate Dehydrogenase |  | 16.9 | 6.0–19.7 | |

Notes Protein: 5.3 mg/ml , Mitochondrial Isolation 2: Protein: 4.4 mg/ml

## 2) Relative Activities (mU/mU CS)

| **Enzyme / Complex** | **Iso 1** | **Iso 2** | **Reference Range** |
| --- | --- | --- | --- |
| Citrate Synthase (CS) |  |  |  |
| Complex I | 0.08 | 0.10 | 0.04–0.12 |
| Complex I+III | 1.02+ | 0.52 | 0.23–0.53 |
| Complex II | 0.28 | 0.57+ | 0.18–0.43 |
| Complex II+III | 0.64 | 1.37+ | 0.29–0.69 |
| Complex III | 2.995 | 5.39+ | 0.72–2.23 |
| Cytochrome c Oxidase (COX) | 1.88 | 2.51+ | 0.90–1.79 |
| Complex V | 1.085+ | 1.45+ | 0.39–0.79 |
| Pyruvate Dehydrogenase |  | 0.0785+ | 0.020–0.050 |

## 3) Relative Activities (mU/mU COX)

| **Enzyme / Complex** | **Iso 1** | **Iso 2** | **Reference Range** |
| --- | --- | --- | --- |
| Citrate Synthase (CS) | 0.53- | 0.40- | 0.56–1.11 |
| Complex I | 0.04 | 0.04 | 0.04–0.11 |
| Complex I+III | 0.55+ | 0.21 | 0.16–0.41 |
| Complex II | 0.15 | 0.23 | 0.12–0.29 |
| Complex II+III | 0.34 | 0.55+ | 0.24–0.52 |
| Complex III | 1.59+ | 2.15+ | 0.61–1.58 |
| Complex V | 0.58+ | 0.58+ | 0.27–0.54 |
| Pyruvate Dehydrogenase |  | 0.031 | 0.018–0.036 |
